# Adaptation to the Local Environment by Modifications of the Photoperiod Response in Crops

**DOI:** 10.1093/pcp/pcu181

**Published:** 2014-11-27

**Authors:** Norihito Nakamichi

**Affiliations:** ^1^Institute of Transformative Bio-Molecules (WPI-ITbM), Nagoya University, Chikusa, Nagoya, 464-8602 Japan; ^2^Division of Biological Science, Graduate School of Science, Nagoya University, Chikusa, Nagoya, 464-8602 Japan; ^3^Precursory Research for Embryonic Science and Technology, Japan Science and Technology Agency, Kawaguchi, Saitama, 332-0022 Japan

**Keywords:** Circadian clock, Flowering time, Post-domestication spread

## Abstract

Flowering plants produce a meristem at the shoot tip where specialized tissue generates shoot apical meristems at the appropriate time to differentiate into reproductive structures, pollinate and efficiently generate seeds. The complex set of molecular and phenological events culminating in development of a flowering meristem is referred to as ‘flowering time’. Flowering time affects plant productivity because plants dedicate energy to produce flowers and seeds rather than vegetative tissue once the molecular decision to initiate flowering has been taken. Thus, initiation of flowering time is an important decision in plants, especially in annual plants including crops. Humans have introduced crops into latitudes and climate areas far from their origin or natural ecosystem, requiring in many cases modification of native flowering times. Recent molecular–genetic studies shed light on the genetic basis related to such introductions. In this review, recent progress regarding crop introductions and their genetic bases are summarized, as well as the potential of other agricultural plants to be introduced into different climatic zones.

## Plants Flower at Appropriate Seasons

**Fig. 1:**
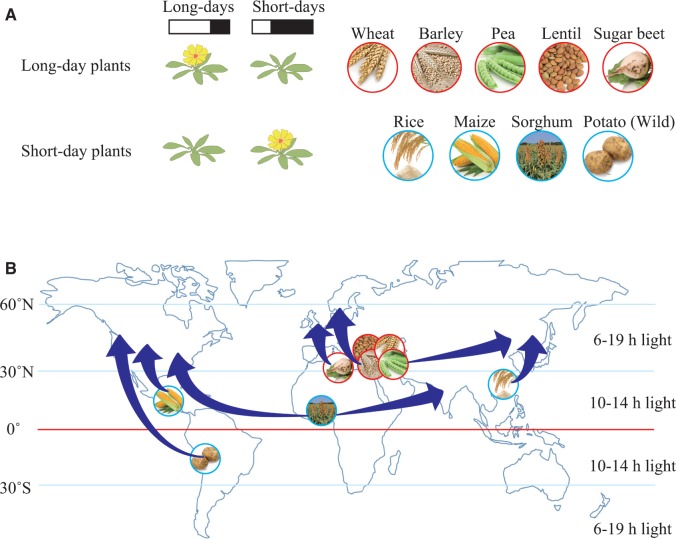
Photoperiodic flowering and post-domestication spread of crops. (A) Photoperiodic flowering of long-day and short-day plants. Long-day crops (wheat, barley, pea, lentil and sugar beet) flower under long days rather than under short days. Short-day crops (rice, maize, sorghum and wild potato) flower more efficiently under short days than under long days. (B) Post-domestication spread of crops. Long-day crops from south-west Asia and the Mediterranean, and short-day crops from south China, Africa, Mexico and the Andes are all grown at higher latitudes. Such spreading is closely associated with altered flowering times.

Grass plants (fam. Poaceae) are generally classified as annual or perennial plants. Perennial plants maintain a continuous vegetative structure for at least 2 years, whereas annual plants complete their life cycle within a year. In order to survive from year to year, annual plants must flower, pollinate and make seeds at the appropriate time by sensing both day-length (or night length) and temperature as seasonal cues. Recent molecular genetic studies have revealed the molecular basis by which grasses sense temperature and day-length changes (Song et al. 2013b). Although there are a few exceptions, long-day grasses generally sense low temperature as a signal to grow in the relatively cold-tolerant vegetative state, a regulatory state known as vernalization. Plants also use changes in day-length information to sense seasons, especially in temperate and cool-temperate zones. Plants that flower in response to lengthening days and when day-length becomes longer than a certain critical length are termed ‘long-day plants’ (Garner and Allard 1920). Long-day grasses include wheat, barley and ryegrass. Other long-day plants include peas, onion, spinach, lettuce, some cruciferous vegetables, sugar beet, carnation and clover (Fig. 1A). Short-day plants are those that regulate flowering based on a long and uninterrupted dark (night) period. These plants include the grasses rice, maize, sorghum and sugar cane, as well as the dicots soybean, strawberry, tobacco, morning glory, squash, green perilla, wild potato and wild tomato (Fig. 1A). Day-neutral plants flower independently of photoperiod (e.g. many cultivars of tomato, potato and eggplant).

## Flowering Time Modification is Related to Local, Regional and Zonal Adaptation

Humans have domesticated long-day and short-day crops, which have in turn bolstered their panglobal migration (Fig. 1B). Seeds of some of the oldest domesticated plants were found in the mortar and mud bricks of ruins in south-west Asia dating from the Early Pre-Pottery Neolithic B era by approximately 10,500–10,100 calibrated years ago (Zohary et al. 2011). There are some common phenotypes among many domesticated plants, a phenomenon known as the ‘domestication syndrome’ (Larson et al. 2014). The domestication syndrome involves a reduction in the dehiscence of seeds, physical and chemical defenses, unnecessary shoots and seed dormancy, but an increase in the predictability of germination and seed size. In addition, domestication coincides with agronomic optimizations such as self-pollination and altered flowering times. These optimizations reduce the energy required to induce seed formation, and enable humans to grow crops at higher or lower latitudes and in diverse climatic regions.

As the cradle of agriculture, south-west Asia had an abundant water supply from snowmelt, and thus large quantities of water could be used for irrigation during the spring and into early summer. However, water was probably in short supply during late summer once mountain run-off had abated. Such a limitation would have forced early farmers to select crops which could be harvested before late summer. These conditions would have enhanced the demand for long-day plants because these plants flower and fruit in response to the long days and short nights of spring, and they would be ready for harvest before late summer droughts. There are six major crops known to have originated in the Fertile Crescent of south-west Asia: emmer wheat, einkorn wheat, barley, lentil, pea and flax (Zohary et al. 2011), all of which are long-day plants. These plants were rapidly spread into neighboring regions on an east–west axis into southern Europe, central Asia and India (Zohary et al. 2011). In contrast, spreading these crops to higher or lower latitudes was relatively difficult because of phenological limitations imposed by differences in temperature and day-length. Selection for earlier or later flowering times would have enabled humans to spread these plants both north and south (Fig. 1B).

Short-day crops are thought to have originated from tropical through low-temperate areas. Sorghum from Africa, Japonica rice from southern China, and maize, potato and eggplant from Mesoamerica and the Andes are presently grown at higher latitudes where summer days are longer (Fig. 1B). These regions are also characterized by drastically lower temperatures after late summer, including frosts. Other than maize, which has the unusual characteristic of seed filling throughout its development, there may not be enough time for seed maturation in short-day plants in high-latitudes or regions of high elevation. Therefore, flowering induction despite long-day conditions is an important agricultural trait in these plants. Indeed, in contrast to wild varieties, domesticated high-latitude cultivars of tomato, potato, sorghum and rice have lost photoperiodicity and easily flower under long-day conditions (Fig. 1B).

Loss of a vernalization requirement by wheat and barley enabled humans to sow in early spring, which decreased damage from snow or frost (Turner et al. 2005). Therefore, loss of vernalization contributed to dispersal of these crops into regions with severe winters. For successful vernalization, barley and wheat employ the genes *VRN1* and *VRN2*, which encode a MADS-box transcription factor and a CCT (for CONSTANS, CONSTANS-LIKE 1 and TIMING OF CAB EXPRESSION 1) domain protein, but they are not conserved in Arabidopsis (Yan et al. 2003, Yan et al. 2004). Mutations of these genes changed these crops from a winter to spring habit, which enables high-latitude growth. Natural variation of *Arabidopsis thaliana FLOWERING LOCUS C* (*FLC*) and *FRIGIDA* (*FRI*) apparently contributed to dissemination of this non-agronomic plant (Shindo et al. 2005, Shindo et al. 2006). *FRI* and *FLC* functions help to explain flowering by vernalization and also the life cycle of Arabidopsis in its many ecological adaptations (Aikawa et al. 2010, Satake 2010, Song et al. 2013a), but these genes are not conserved in other long-day plants such as wheat and barley (Ream et al. 2012). These lines of study suggest that vernalization systems evolved independently in different plant groups (Ream et al. 2012).

Recent progress in genomic studies has revealed that genetic modification of the photoperiodic pathway may also have contributed to the north–south spreading of long-day plants. In addition, changing the photoperiodic flowering response of short-day plants is also associated with dissemination into higher latitudes. This mini review illustrates possible genetic modifications in long-day and short-day crops for spreading into different climatic areas from their geographic origins.

## Molecular Basis of Photoperiodic Flowering in a Model Long-Day Plant

**Fig. 2:**
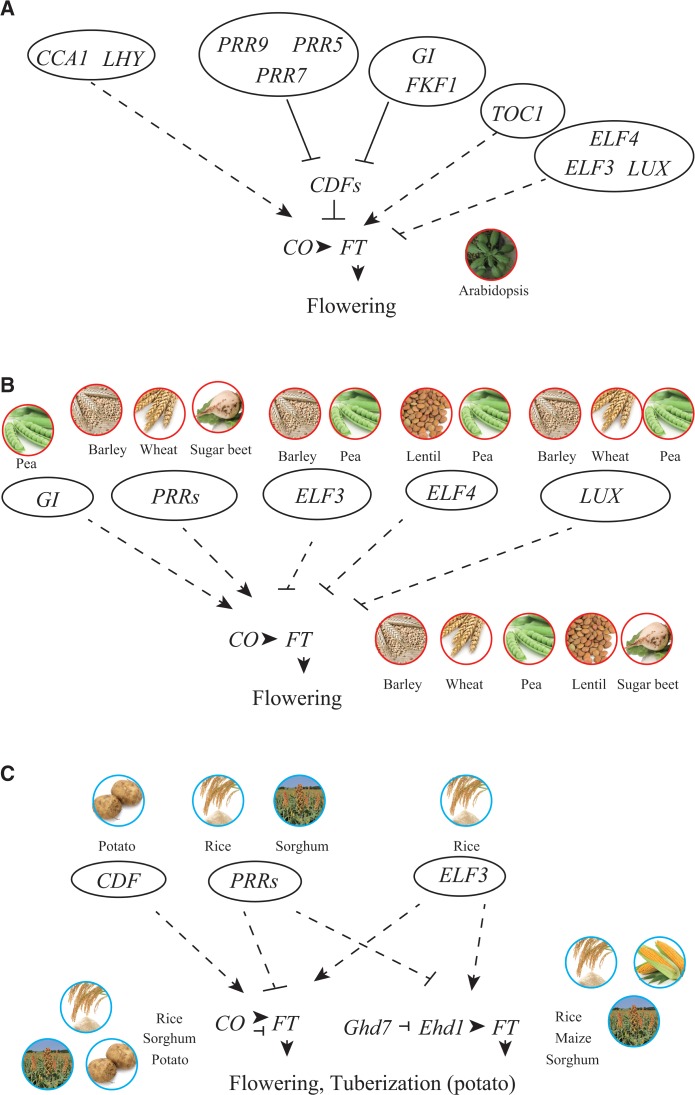
Genes involved in photoperiodic flowering time regulation in some crops. (A) Genes involved in photoperiodic flowering regulation in the long-day plant Arabidopsis. Solid and dashed lines indicate direct and indirect regulation, respectively. (B) Genes involved in photoperiodic flowering in long-day crops. (C) Genes involved in photoperiodic flowering in short-day crops. In rice, CO up-regulates *FT* under long days, but down-regulates it under short days.

Variations of *FLC* and *FRI* probably contributed to the geographic spread of Arabidopsis. In addition, natural variation of the photoperiodic gene *CONSTANS* (*CO*) also contributed (Rosas et al. 2014). The molecular genetics of photoperiodic flowering time have been well studied in the long-day model plant Arabidopsis (Fig. 2A). Arabidopsis plants are relative long-day plants, and flower earlier under long-day conditions than under short-day conditions. Forward and reverse genetics have been used to determine that this plant has genes involved both in repressing and in accelerating photoperiodic flowering time. *CO*, *GIGANTEA* (*GI*), *FLAVIN-BINDING*, *KELCH REPEAT*, *F BOX 1* (*FKF1*), *PSEUDO-RESPONSE REGULATOR 9* (*PRR9*), *PRR7*, *PRR5*, *CASEIN KINASE 2 β subunit* (*CKB3*), *NIGHT LIGHT-INDUCIBLE AND CLOCK REGULATED GENES 1* (*LNK1*), *LNK2* and *FT*, encoding a florigen, are activators of the photoperiodic flowering time pathway (Putterill et al. 1995, Fowler et al. 1999, Kardailsky et al. 1999, Kobayashi et al. 1999, Sugano et al. 1999, Nelson et al. 2000, Mizoguchi et al. 2005, Nakamichi et al. 2007, Song et al. 2012, Rugnone et al. 2013). *CYCLING DOF FACTOR* (*CDF*), *CIRCADIAN CLOCK-ASSOCIATED 1* (*CCA1*), *LATE ELONGATED HYPOCOTYL* (*LHY*), *TIMING OF CAB EXPRESSION 1* (*TOC1/PRR1*), *REVEILLE8* [*RVE8/LHY-CCA1-LIKE 5* (*LCL5*)], *FIONA1* (*FIO1*), *LIGHT-REGULATED WD1* (*LWD1*), *LWD2*, *EARLY FLOWERING* (*ELF3*), *ELF4* and *LUXARRHYTHMO* [*LUX*/*PHYTOCLOCK 1* (*PCL1*)] are repressors for photoperiodic flowering time (Hicks et al. 2001, Doyle et al. 2002, Yanovsky and Kay 2002, Hazen et al. 2005, Imaizumi et al. 2005, Mizoguchi et al. 2005, Onai and Ishiura 2005, Kim et al. 2008, Wu et al. 2008, Fornara et al. 2009, Farinas and Mas 2011, Rawat et al. 2011, Song et al. 2012). Of these genes, *PRR9*, *PRR7*, *PRR5*, *TOC1*, *GI*, *CCA1*, *LHY*, *CKB3*, *RVE8*, *ELF3*, *ELF4*, *LUX*, *FIO1*, *LNK1*, *LNK2*, *LWD1* and *LWD2* are thought to be the circadian clock components that generate internal timekeeping by which the plant can be regulated by reference to external time (Nakamichi 2011, McClung 2014). Generally, it is thought that the clock generates certain ‘photo-sensitive’ and ‘dark-sensitive’ phases in a day–night cycle (Bunning 1967). The plant is induced to make flowers if a light signal inputs to the plant during the photo-sensitive phase. However, if the light signal inputs during a dark-sensitive phase, the plant produces fewer flowers. This mechanism is called the external coincidence model. This model was validated by a molecular study (Yanovsky and Kay 2002), but recent molecular evidence suggests that the exact mechanism for photoperiodic flowering time is more complicated. In Arabidopsis, *FT* is transcribed more under long days than under short days depending on *CO* (Kardailsky et al. 1999, Kobayashi et al. 1999). External coincidence was proposed to be due to *CO* expression timing and its stabilization or degradation by the light signal (Valverde et al. 2004). *CO* is expressed 10–14^h after ‘light on’, and CO protein is stabilized by far-red and blue light, but degrades under dark and red light conditions (Valverde et al. 2004). CONSTITUTIVE PHOTOMORPHOGENIC 1 degrades CO in the dark (Jang et al. 2008, Liu et al. 2008). In contrast, blue light photoreceptor FKF1 and its partner GI stabilize CO in blue light (Song et al. 2012). FKF1 and GI protein expression is coincident in the afternoon under long-day conditions. However, under short-day conditions, FKF1 and GI expression phases do not align. These results suggest that the coincidence between FKF1 and GI that occurs under long-day conditions is also crucial to the photoperiodic flowering pathway (Sawa et al. 2007). FKF1 and GI degrade CDF1 and its homologs (CDF2, CDF3 and CDF5), proteins that encode transcriptional repressors for *CO* and *FT* (Imaizumi et al. 2005, Fornara et al. 2009, Song et al. 2012). *CDF1* is also regulated at the transcriptional level by clock-associated transcription factors known as PRRs. ChIP-seq analyses of PRR5–GFP (green fluorescent protein) and PRR7–HA (hemagglutinin) demonstrated that PRR proteins associate with upstream regions of *CDF* genes (Nakamichi et al. 2012, Liu et al. 2013). In *prr9 prr7*, *prr7 prr5* and *prr9 prr7 prr5* double and triple mutants, *CDF1* expression is up-regulated (Nakamichi et al. 2007). Peak levels of *CDF1* expression are not changed in these *prr* mutants, but trough levels of *CDF1* are up-regulated (Nakamichi et al. 2007). These results indicate that the clock mechanism regulates not only the expression phase of *CDF1*, but also expression amplitude. Collectively, these studies revealed a molecular mechanism underlying the photoperiodic flowering time pathway. Because homologs of these genes are found in many plants, it should be possible to extrapolate from what is known about clock genetics in Arabidopsis to other plants.

## Post-Domestication Spread by Altering Photoperiodism of Barley

Modern cultivars of long-day plants arose in the Fertile Crescent, but modern cultivars have different flowering times compared with their ancestors. The original cultivars of barley were sown in autumn, germinated and grew over the winter, and flowered during the next spring, indicative of the so-called winter growth habit (Zohary et al. 2011). Long-term vernalization would thus prevent flowering of long-day plants prematurely during the autumn even if day-length were longer than the critical flowering-inducible day-length threshold. According to this life cycle, early barley cultivation was adapted for the local environment. However, in more northern areas such as Europe and the Caucasus, sowing barley in autumn was unsuitable because young seedlings would be damaged by winter frosts and snow. This situation may have precipitated the selection of cultivars that had lost the vernalization requirement, enabling early spring sowing once the danger of frost was over. This selection unintentionally caused reduction in the vegetative growth phase because of the shorter period between germination and flowering, and sequentially decreased yield. However, among vernalization-independent cultivars, higher yield cultivars could be selected for delayed flowering time (Turner et al. 2005). These cultivars extended the vegetative growth phase, providing additional biomass for photosynthetic activity and increased yield. A genetic study identified a responsible gene, *Photoperiod-H1* (*Ppd-H1*), for flowering time regulation (Turner et al. 2005). *ppd-H1* alleles flower late, probably though decreased expression of *HvFT2*, a homolog of Arabidopsis *FT*. In much of North America and Western Europe, *ppd-H1* late-flowering alleles are mainly used (Turner et al. 2005). *Ppd-H1* encodes a pseudo-response regulator. In Arabidopsis, four *PRR* genes are implicated in clock function, but expression of clock-controlled genes (e.g. *HvGI* and *HvTOC1*) is similar for both *ppd-h1* and *Ppd-H1* alleles, indicating that *Ppd-H1* is not involved in the clock system (Campoli et al. 2012).

Soon after J.H. Muller established the concept in *Drosophila* that ionizing irradiation causes mutations, induced mutations were reported in barley (Zakhrabekova et al. 2012). The Swedish Seed Association selected early-flowering cultivars from this mutant collection, including the *praematurum-a* (*mat-a*) mutants. These mutants were allelic to *erectoides-o.16* and *early maturity 8* (*eam8*) mutants, some of which occurred naturally in Japan and Russia (Zakhrabekova et al. 2012). These early-flowering cultivars have since been used in regions having short growing seasons, such as Scandinavia and Iceland. Early-flowering cultivars were also introduced near the Equator because farmers can harvest grains several times a year (Zakhrabekova et al. 2012). Recent genetic studies identified the gene responsible for this early flowering as a homolog of Arabidopsis *ELF3* (Faure et al. 2012, Zakhrabekova et al. 2012). In *eam8* and *mat-a* mutants, diurnal and circadian expression patterns of clock-associated genes (e.g. *HvCCA1*, *HvTOC1* and *HvGI*) and photoperiod pathway genes (*Ppd-H1*, *HvCO1* and *HvFT1*) are drastically changed, indicating involvement of *HvELF3* in the clock system (Faure et al. 2012, Zakhrabekova et al. 2012).

*eam10* is an early-flowering cultivar which was created by X-ray irradiation (Campoli et al. 2013). It flowers earlier than its parental plant under both short days and long days. A candidate gene for the *eam10* locus was identified by high-throughput DNA sequencing as an Arabidopsis *LUX* homolog (Campoli et al. 2013). Expression of clock-associated genes was altered in *eam10* compared with the parental plants, indicating that *Eam10* functions in the clock. It is also known that *Eam10* and *Ppd-H1* genetically interact with each other because the *eam10* mutation in a *Ppd-H1* genetic background results in very early flowering (∼30 d after sowing) even under short-day conditions (Campoli et al. 2013). *eam10*/*Ppd-H1* may have utility in some very short-day geographic regions. In the case of barley, both late- and early-flowering cultivars have been selected for different reasons.

## Post-Domestication Spread by Altering Photoperiodism of Wheat

Wheat is a long-day plant, but photoperiod insensitivity is an important part of its Green Revolution modifications (Beales et al. 2007). Photoperiod insensitivity in wheat is generally considered to be early flowering in response to short days. The semi-dominant *Photoperiod-D1a* (*Ppd-D1a*) allele is one characteristic trait of photoperiod-insensitive cultivars. The *Ppd-D1a* early-flowering allele gives high yields in southern European countries on the Balkan peninsula, but low yield in Britain (Worland et al. 1998). Early flowering associated with high yields in southern Europe is probably due to the development of mature seed before the onset of high summer temperatures. It is believed that the *Ppd-D1a* allele was introduced into European wheat from the Japanese variety Akakomugi, by Strampelli’s breeding program of the early 20th century (Beales et al. 2007). It is now known that this early flowering is associated with deletion of the upstream region of a *PRR* gene (Beales et al. 2007). Faulty expression of *PRR* causes up-regulation of *TaFT1*, resulting in similar activity of *PRR* in regulating flowering time in Arabidopsis. Expression of *TaGI*, which is controlled by the clock, is not changed in *Ppd-D1a*, indicating that this gene is not involved in the clock (Beales et al. 2007).

Einkorn wheat (*Triticum monococcum* L.) is a wild progenitor species of wheat. Because einkorn wheat is diploid, it may be a model species for understanding the genetic basis for any biological processes in wheat, whose gametes are triploid. Early-flowering alleles were created in einkorn wheat by X-ray irradiation (Shindo and Sasakuma 2001), and a recent genetic study suggests that a homolog of Arabidiosis *LUX* is the best candidate of the early-flowering allele KT3-5 (Mizuno et al. 2012). In KT3-5, expression of clock-associated genes and photoperiod flowering genes differs from that of parental plants, suggesting that *LUX* in this species is involved in clock function.

## Post-Domestication Spread of Sugar Beet

An intriguing case of selecting for a late-flowering cultivar in long-day plants was reported in sugar beet (Pin et al. 2012). One wild sea beet (*Beta vulgaris maritima*) is an annual, long-day plant, but sugar beets (ssp. *vulgaris*) and sea beets (ssp. *maritima*) in northern Europe are biennials, and require vernalization followed by long days for initiating flowering. The *BOLTING TIME CONTROL1* (*BvBTC1*) gene is involved in this annual and biennial control. Annual beet varieties have a functional *BvBTC1* gene, but biennial beets have lost this activity. Loss of *BvBTC1* function results in late flowering and introduces a vernalization requirement to flower, resulting in the biennial life cycle. Biennial beet produces a large biomass, including a big root. *BvBTC1* encodes a pseudo-response regulator, and non-functional alleles of *BvBTC1* decrease *BvFT* gene expression. Expression of homologous genes involved in the Arabidopsis circadian clock (e.g. *BvLHY*, *BvTOC1*, *BvPRR7* and *BvGI*) is the same in both annual and biennial beets, suggesting that *BvBTC1* operates independently from the clock system (Pin et al. 2012).

## Post-Domestication Spread by Altering Photoperiodism of Pea

The legumes fall into two distinct clades. One includes the long-day plants (e.g. lotus, lentil, pea and chickpea), originally from temperate zones, and the other includes short-day plants (bean, soybean and cowpea) from lower latitudes (Hecht et al. 2011). Garden pea is a model legume plant whose genetic basis for photoperiodic flowering has been studied. There are four natural variations in domesticated pea that differ from ancestral flowering control (Weller et al. 1997): *SN* (sterile nodes), *LF* (late flowering), *HR* (high response) and *E* (early initiating). It is thought that pre-domestication peas carrying *HR* were near-obligate long-day flowering, and thus had a typical winter habit in which pea germinates in the autumn and flowers during the next spring (Abbo et al. 2003). Many domesticated cultivars carry the *hr* mutation, which allows pea to grow at higher latitudes, and which is associated with the spring blossom habit. This mutation causes flowering during short days. *HR* is an ortholog of Arabidipsis *ELF3* (Weller et al. 2012). Circadian gene expression in *hr* plants is altered, and *HR* complements Arabidopsis *elf3* mutants, indicating that *HR* acts in the clock. Lentil pea was also domesticated in south-west Asia and its ancestor is a long-day plant. Domesticated lentils could be spread to regions with short day-length and drought by reducing photoperiod sensitivity, thereby allowing early flowering (Weller et al. 2012). As in pea, mutation of the *ELF3* homolog was found in early flowering cultivars, suggesting that *ELF3* mutations were crucial for spreading lentils (Weller et al. 2012). Pea *SN* was identified as an ortholog of Arabidopsis *LUX* (Liew et al. 2014). Plants carrying *sn* flower early under short days, and have altered circadian rhythms, indicating that *SN* is involved in clock regulation. It was also demonstrated that one major *sn* allele is used in Alaska, where the summers are short and peas cannot survive the overwintering habit (Liew et al. 2014).

Artificial (induced) mutagenesis approaches generated early-flowering alleles *DNE* (*die neutralis*) and *PPD* (*photoperiod*), and a late-flowering allele *LATE1* (*LATE BLOOMER1*). Although no corresponding gene for *PPD* has been revealed, *DNE* was identified as an ortholog of Arabidopsis *ELF4* (Liew et al. 2009) and *LATE* as an ortholog of Arabidopsis *GI* (Hecht et al. 2007). *SN* is epistatic to *LATE1* for flowering initiation (Hecht et al. 2007). *DNE* and *LATE1* genetically interact with each other in that *dne late1* double mutants flower earlier than *late1* but later than *dne* (Liew et al. 2009).

## Possible Common Genetic Factors Implicated in Flowering in Long-Day Plants

Genetic studies focusing on the relationship between photoperiodic flowering and post-domestication spread in the long-day plants barley, wheat, pea, lentil and sugar beet found that mutations implicated in regulation of the *CO–FT* module for flowering time were related to geographic dispersal (Fig. 1B). Mutations in *ELF3*, *ELF4* and *LUX* result in early flowering and were selected for planting where the summers are short or very dry. Mutations in *GI* and *PRR* genes result in late flowering. There is strong selective pressure for *PRR* mutants because late flowering causes extension of the vegetative phase resulting in increased final biomass (Turner et al. 2005, Pin et al. 2012). *ELF3*, *ELF4*, *LUX* and *GI* are involved in the clock in these plants, whereas *PRR* is not. *PRR* probably preferentially regulates the flowering pathway rather than the clock. Aberrant expression of *PRR* in wheat also causes early flowering (Beales et al. 2007), suggesting that quantitative control of *PRR* may correspondingly control flowering time in many long-day plants.

## Post-Domestication Spread by Altering Photoperiodism of Rice

Extensive quantitative trait locus (QTL) studies in rice have identified genes implicated in photoperiodic flowering time and revealed some molecular mechanisms for flowering time regulation (Yamamoto et al. 1998, Yano et al. 2000, Kojima et al. 2002, Doi et al. 2004, Xue et al. 2008, Matsubara et al. 2012, Saito et al. 2012, Fujino et al. 2013, Koo et al. 2013, Yan et al. 2013). *Heading date 1* (*Hd1*) encodes a homolog of Arabidopsis *CO* (Yano et al. 2000). *Hd1* induces flowering under short days but represses flowering under long days through regulation of *Hd3a*, a homolog of Arabidopsis *FT* (Kojima et al. 2002, Hayama et al. 2003). Other *Hd* genes also regulate flowering time through *Hd3a* expression. According to an analysis of polymorphisms in some *Hd* genes, rice cultivars were selected whose flowering times were locally appropriate. Cultivars having double non-functional alleles of *hd2/prr37* and *hd4/ghd7* (*Grain number, Plant height and Heading date 7*) are used at latitudes higher than 40°N (Koo et al. 2013, Yan et al. 2013). These cultivars flower very early under long-day conditions. This property allowed harvesting before cool autumn temperatures inhibited grain maturation. Polymorphisms within the promoter region of *Hd3a* and coding region of *Hd1* also influence flowering time, and might contribute to local adaptation (Takahashi et al. 2009). Non-functional alleles of *RFT1* associated with late flowering were found in Indica rice at peri-equatorial latitudes. Late flowering might be beneficial in rice grown under specific environment conditions such as seasonal rapid flooding (Ogiso-Tanaka et al. 2013). On the other hand, functional *RFT* is required for early flowering under long-day conditions in summer in temperate zones (Itoh and Izawa 2013). An *Ehd1* mutant called *ef1* grown in Taiwan has an extended vegetative growth phase (Inoue et al. 1998, Saito et al. 2009). Mutant alleles *hd16*, *hd17* and *hd5* are found in varieties cultivated on the island of Hokkaido in Japan, and have probably been selected due to demands for flowering time in these specific locations (Matsubara et al. 2012, Fujino et al. 2013, Hori et al. 2013).

Studies in rice have also provided the molecular mechanism underlying photoperiodic flowering (Itoh and Izawa 2013). As mentioned above, florigen expression at the appropriate day-length is important as a seasonal response. In Arabidopsis, the mechanism by which florigen is induced by *CO* in the late afternoon under long-day conditions is crucial for clock-dependent photoperiodic flowering. In rice, *Hd1* suppresses *Hd3a* expression under short days (Hayama et al. 2003). In addition, the gating of two regulators, *Ehd1* (*Early heading date 1*) and *Ghd7*, is crucial for measuring precise day-length. *Ehd1* encodes a B-type response regulator and induces *Hd3a* expression independently of *Hd1* (Doi et al. 2004). *Ghd7* encodes a CCT domain protein and represses *Ehd1* (Xue et al. 2008). Ghd7 is a structural homolog of wheat VRN2. Both *Hd1*-dependent and *Ehd1*-dependent *Hd3a* regulation are crucial for photoperiodic flowering in rice (Itoh and Izawa 2013).

In addition, genetic studies identified the *ELF3* homologs for *Early flowering 7* (*Ef7*)/*Hd17* (Matsubara et al. 2012, Saito et al. 2012). *Ef7* mutations resulted in late flowering, but this phenotype is suppressed by *ghd7*, indicating that *OsELF3* regulates flowering though *Ghd7* (Saito et al. 2012). Circadian gene expression is altered in *oself3-1* mutants compared with the parental plants, suggesting that *OsELF3* is involved in the clock (Zhao et al. 2012, Y. Yang et al. 2013).

## Post-Domestication Spread by Altering Photoperiodism of Sorghum

Sorghum [*Sorghum bicolor* (L.) Moench], a C_4_ plant, was originally cultivated in Africa and has provided a robust food source (Larson et al. 2014). In addition to its cultivation as a food crop (grain sorghum), sorghum is also used as an energy crop that provides lignocellulosic-based biofuels (sweet sorghum). Delayed flowering causes extension of the vegetative growth phase with a concomitant increase in biomass. Delayed flowering variants were selected for use in biofuel sorghum cultivation but, in the case of sorghum as a food crop, early-flowering cultivars were useful because there needed to be enough time for grain maturation before the onset of unfavorable hot and dry weather conditions. Therefore, quite different agronomic demands can be expected to determine selection for flowering time, which often needs to be precisely controlled (Murphy et al. 2011).

Human selection activity in sorghum has resulted in a wide variety of photoperiodic flowering times (Murphy et al. 2014). It had been suggested that grain sorghum was cultivated in tropical zones originally as short-day plants. Harvesting sorghum grain in temperate zones was difficult because sorghum was a short-day plant and did not flower under long-day conditions during summer. Photoperiod-insensitive cultivars of grain sorghum (*ma1* and *ma6* recessive alleles) were thought to be selected in South Africa or Egypt, where day-lengths were too long, so that day-length-sensitive sorghum could not flower (Murphy et al. 2014). These cultivars were then introduced into North America. Using these early flowering grain sorghums as a genetic foundation, modern sweet sorghums were then generated. However, breeders in the USA started to generate late-flowering sweet sorghum by crossing early-flowering sweet sorghum and late-flowering grain sorghum, because late flowering increases the yield of sweet sorghum (Murphy et al. 2014).

The photoperiod-insensitive cultivar *ma1 ma6* can flower and produce grains even under long-day conditions such as during summers in temperate zones (Murphy et al. 2011). The *Ma1* locus has been identified as *SbPRR37* (Murphy et al. 2011). *ma1* flowers early under long-day conditions due to higher expression of *FT* genes via altered expression of *SbCO* and *SbEhd1*. Interestingly, *PRR37* expression itself showed a dual peak pattern under long-day conditions. The peaks of *PRR37* occur during early morning and early evening under long days, but night-time expression of *PRR37* is diminished by short days as well as signaling due to darkness. Diminished *PRR37* expression during the evening results in decreased PRR37 flowering-repressor activity, permitting floral initiation. Collectively, the coincidence between *PRR37* expression and darkness probably triggers the repression of flowering time (Murphy et al. 2011). *Ma6* is a strong flowering repressor. A recent study identified *Ma6* as a CCT domain-containing protein similar to rice *Ghd7* (Murphy et al. 2014). Expression of *Ma6* is controlled by both the clock and photoperiod systems, and peaked both in the morning and during the early night-time under long days. Night-time expression of *Ma6* was diminished under short-day conditions. Collectively, *Ma6* expression during the night-time is important for delaying flowering under long days (Murphy et al. 2014).

## Post-Domestication Spread of Maize

Maize was domesticated in southern Mexico from its progenitor teosinte. Flowering of teosinte and maize grown in tropical zones is repressed under long days, whereas maize grown in temperate zones has decreased photoperiod sensitivity and flowers under long-day conditions. There are no major QTLs between B73, an important founder line in the USA, and 25 world-wide cultivars (Buckler et al. 2009). Genome-wide association studies using B73 and 25 world-wide cultivars as parents (Hung et al. 2012) or using teosinte as one parent revealed that *ZmCCT* is responsible for delaying flowering under long-days in maize (Q. Yang et al. 2013). *ZmCCT* is a structural homolog of rice *Ghd7*, and is responsible for photoperiodic flowering regulation, which is attenuated in temperate maize (Hung et al. 2012). Furthermore, a CACTA-like transposable element (TE) is inserted upstream of *ZmCCT* along with single nucleotide polymorphisms (SNPs) in inbred lines carrying a non-functional *ZmCCT* (Q. Yang et al. 2013). Insertion of the CACTA-like TE upstream of *ZmCCT* enhances cytosine methylation in the upstream regulatory region of the gene and results in suppression of *ZmCCT* (Q. Yang et al. 2013). Non-functional *ZmCCT* alleles were found not only in inbred temperate zone maize, but also in those adapted for tropical zones, suggesting that *ZmCCT* alleles were selected at a relatively early phase after domestication. Since the early flowering effect of *ZmCCT* alleles would not be apparent under short days in tropical zones, any other effects of selection of non-functional *ZmCCT* alleles should be hidden. A hint about one of these reasons might come from rice, where a mutation of *Ghd7* altered grain architecture (Hung et al. 2012).

## Post-Domestication Spread by Altering Photoperiodism of Potato

Potato was domesticated on the equatorial highlands of South America. Wild potato varieties of the Andean region (*Solanum tuberosum* spp. *andigena*) make tubers underground under short days but not under long-day conditions, meaning that the tuber formation response is strictly dependent on day-length (Rodriguez-Falcon et al. 2006). However, modern potato cultivars in higher latitudes can make tubers under long days. The *CO–FT* module regulates not only photoperiodic flowering but also tuber formation (Rodriguez-Falcon et al. 2006). A recent genetic study revealed that a homolog of Arabidopsis *CDF1* (*StCDF1*) is a major regulator of photoperiodic tuber formation, and that this mutation is associated with post-domestication spread (Kloosterman et al. 2013). Two variants (*StCDF1.2* and *StCDF1.3*) were found in early-maturation cultivars, and a third (*StCDF1.1*) in a very late-maturation cultivar. *StCDF1.1* encodes the full length of StCDF1, whereas *StCDF1.2* and *StCDF1.3* encode truncated forms without the C-terminal region. The StCDF1.1 C-terminal region has the capacity to bind StFKF1. Because AtFKF1 binds to AtCDF1 for degradation (Imaizumi et al. 2005), it is not surprising that a variant form lacking this region is more stable than StCDF1.1 (Kloosterman et al. 2013). In addition, overexpression of *StCDF1.2* results in early tuberization as well as early flowering, indicating that *StCDF1* regulates these photoperiodic regulations.

## Common Genetic Factors Implicated in Flowering in Short-Day Plants

Dissemination of domesticated crops on a north–south axis was dependent on human selection for mutations implicated in temporal regulation of the flowering pathway in short-day plants such as rice, sorghum, maize and potato. Short-day plants probably use two regulatory modules (CO–FT, and Ghd7–Ehd1–FT) to induce flowering, one of which (Ghd7–Ehd1–FT) is found in short-day plants, but not yet in long-day plants (Fig. 1C). It has also been suggested that the regulation polarities of proteins upstream of CO–FT are inverse to those in long-day plants. For instance, *ELF3* and *CDF1* are activators, and *PRR* genes are repressors. One explanation of such an inverse arrangement is that CO in short-day plants serves as a repressor for *FT* under long-day conditions, but in long-day plants CO acts as an activator (Hayama et al. 2003, Kloosterman et al. 2013), but additional possibilities remain to be explored.

## Perspectives

Dispersion of a few key crops was closely associated with pre-historic human migration from the three cradles of agriculture. Thanks to an impressive array of genetic studies, it is now clear that clock-associated genes were important contributors to early selection events, and are now intriguing targets for changing flowering time in any annual plant to allow expansion of cultivation regions. In addition, industrialized societies produce social demands on agricultural production, which did not occur before. For example, there are expectations for variety and plenty in the food supplies of wealthy countries, even if these products are difficult to grow, are out of season or can only be grown far away. Some of these plants can be grown throughout the year in greenhouses or by diverting low-latitude arable areas, but the cost of these practices is relatively high and increases the carbon footprint of special-commodity agriculture. In addition, flowering times will continue to need optimization because the global climate is changing, but the day-length conditions for a given latitude are not. If summer temperatures in temperate zones continue to increase, harvests will need to be advanced because some developmental processes (mainly pollen, and flower and seed maturation) are sensitive to high temperatures. Humans have expanded cultivation areas by advancing or delaying flowering times, but this selection was mainly focused on a few crops. However, there are other agricultural products whose flowering times have not been optimized. To this end, we have to understand the genetic basis for controlling flowering pathways both in long-day and in short-day plants.

Given that a few common genetic mutations were selected for altering flowering time across a number of crops, it is not so difficult to imagine a general genetic basis of flowering time in many plants (Fig. 2). There are two types of *PRR* genes, one of which is a flowering accelerator and the other is a repressor. Both types of *PRR* genes regulate *FT* expression, but flowering accelerator *PRR* genes are found in long-day plants, whereas flowering repressor *PRR* genes are in short-day plants. In the model long-day plant Arabidopsis, the *PRR* gene family is involved in the circadian clock system, and also directly regulates expression of *CDF* genes (Nakamichi et al. 2012, Liu et al. 2013), encoding transcriptional repressors for *CO* and *FT* (Song et al. 2012). Although only Arabidopsis and potato *CDF* are repressors of *CO*, these *CDF* genes may be essential mediators between the clock and florigen production. *CDF* genes in long-day Arabidopsis are repressors of flowering, but in short-day potato they act as accelerators, probaby due to opposite polarity of *CO* function between short-day and long-day plants. In addition to *CO*, short-day plants employ a CCT protein (Ghd7 in rice and ZmCCT in maize)–Ehd1 module. This module provides rice with a stringent recognition of day-length (i.e. to within 30 min) (Itoh et al. 2010). Because the range of day-length changes is smaller in lower latitudes than in higher latitudes, such accurate day-length sensitivity may be required for flowering at specific seasons in plants originally grown in lower latitudes. Studies on the relationship between these upstream modules (CDF–CO–FT and Ghd7–Ehd1–FT) seem important for understanding flowering time quantitatively in plants originally grown in lower latitudes.

## Funding

Preparation of this review was supported by the Japan Science and Technology Agency [Precursory Research for Embryonic Science and Technology Grant 20109]; the Ministry of Education, Culture, Sports, Science, and Technology [Grants-in-Aid No. 26870267].

## Abbreviations

CCT: CONSTANS, CONSTANS-LIKE1 and TOC1
CDF: CYCLING DOF FACTOR
‘clock’: circadian clock
CO: CONSTANS
Ehd1: Early heading date 1
ELF: EARLY FLOWERING
FKF1: FLAVIN-BINDING, KELCH REPEAT, F BOX 1
Ghd7: Grain number, Plant height and Heading date 7
GI: GIGANTEA
Hd: Heading date
LUX: LUXARRHYTHMO
PPD: PHOTOPERIOD
PRR: PSEUDO-RESPONSE REGULATOR
QTL: quantitative trait locus
TOC1: TIMING OF CAB EXPRESSION 1

## References

[pcu181-B1] Abbo S, Shtienberg D, Lichtenzveig J, Lev-Yadun S, Gopher A (2003). The chickpea, summer cropping, and a new model for pulse domestication in the ancient near east. Q. Rev. Biol..

[pcu181-B2] Aikawa S, Kobayashi MJ, Satake A, Shimizu KK, Kudoh H (2010). Robust control of the seasonal expression of the Arabidopsis FLC gene in a fluctuating environment. Proc. Natl Acad. Sci. USA.

[pcu181-B3] Beales J, Turner A, Griffiths S, Snape JW, Laurie DA (2007). A pseudo-response regulator is misexpressed in the photoperiod insensitive Ppd-D1a mutant of wheat (Triticum aestivum L.). Theor. Appl. Genet..

[pcu181-B4] Buckler ES, Holland JB, Bradbury PJ, Acharya CB, Brown PJ, Browne C (2009). The genetic architecture of maize flowering time. Science.

[pcu181-B5] Bunning E (1967). The Physiological Clock.

[pcu181-B6] Campoli C, Pankin A, Drosse B, Casao CM, Davis SJ, von Korff M (2013). HvLUX1 is a candidate gene underlying the early maturity 10 locus in barley: phylogeny, diversity, and interactions with the circadian clock and photoperiodic pathways. New Phytol..

[pcu181-B7] Campoli C, Shtaya M, Davis SJ, von Korff M (2012). Expression conservation within the circadian clock of a monocot: natural variation at barley Ppd-H1 affects circadian expression of flowering time genes, but not clock orthologs. BMC Plant Biol..

[pcu181-B8] Doi K, Izawa T, Fuse T, Yamanouchi U, Kubo T, Shimatani Z (2004). Ehd1, a B-type response regulator in rice, confers short-day promotion of flowering and controls FT-like gene expression independently of Hd1. Genes Dev..

[pcu181-B9] Doyle MR, Davis SJ, Bastow RM, McWatters HG, Kozma-Bognar L, Nagy F (2002). The ELF4 gene controls circadian rhythms and flowering time in Arabidopsis thaliana. Nature.

[pcu181-B10] Farinas B, Mas P (2011). Functional implication of the MYB transcription factor RVE8/LCL5 in the circadian control of histone acetylation. Plant J..

[pcu181-B11] Faure S, Turner AS, Gruszka D, Christodoulou V, Davis SJ, von Korff M (2012). Mutation at the circadian clock gene EARLY MATURITY 8 adapts domesticated barley (Hordeum vulgare) to short growing seasons. Proc. Natl Acad. Sci. USA.

[pcu181-B12] Fornara F, Panigrahi KC, Gissot L, Sauerbrunn N, Ruhl M, Jarillo JA (2009). Arabidopsis DOF transcription factors act redundantly to reduce CONSTANS expression and are essential for a photoperiodic flowering response. Dev. Cell.

[pcu181-B13] Fowler S, Lee K, Onouchi H, Samach A, Richardson K, Morris B (1999). GIGANTEA: a circadian clock-controlled gene that regulates photoperiodic flowering in Arabidopsis and encodes a protein with several possible membrane-spanning domains. EMBO J..

[pcu181-B14] Fujino K, Yamanouchi U, Yano M (2013). Roles of the Hd5 gene controlling heading date for adaptation to the northern limits of rice cultivation. Theor. Appl. Genet..

[pcu181-B15] Garner WW, Allard HA (1920). Effect of the relative length of day and night and other factors of the environment on growth and reproduction in plants. J. Agric. Res..

[pcu181-B16] Hayama R, Yokoi S, Tamaki S, Yano M, Shimamoto K (2003). Adaptation of photoperiodic control pathways produces short-day flowering in rice. Nature.

[pcu181-B17] Hazen SP, Schultz TF, Pruneda-Paz JL, Borevitz JO, Ecker JR, Kay SA (2005). LUX ARRHYTHMO encodes a Myb domain protein essential for circadian rhythms. Proc. Natl Acad. Sci. USA.

[pcu181-B18] Hecht V, Knowles CL, Vander Schoor JK, Liew LC, Jones SE, Lambert MJ (2007). Pea LATE BLOOMER1 is a GIGANTEA ortholog with roles in photoperiodic flowering, deetiolation, and transcriptional regulation of circadian clock gene homologs. Plant Physiol..

[pcu181-B19] Hecht V, Laurie RE, Vander Schoor JK, Ridge S, Knowles CL, Liew LC (2011). The pea GIGAS gene is a FLOWERING LOCUS T homolog necessary for graft-transmissible specification of flowering but not for responsiveness to photoperiod. Plant Cell.

[pcu181-B20] Hicks KA, Albertson TM, Wagner DR (2001). EARLY FLOWERING3 encodes a novel protein that regulates circadian clock function and flowering in Arabidopsis. Plant Cell.

[pcu181-B21] Hori K, Ogiso-Tanaka E, Matsubara K, Yamanouchi U, Ebana K, Yano M (2013). Hd16, a gene for casein kinase I, is involved in the control of rice flowering time by modulating the day-length response. Plant J..

[pcu181-B22] Hung HY, Shannon LM, Tian F, Bradbury PJ, Chen C, Flint-Garcia SA (2012). ZmCCT and the genetic basis of day-length adaptation underlying the postdomestication spread of maize. Proc. Natl Acad. Sci. USA.

[pcu181-B23] Imaizumi T, Schultz TF, Harmon FG, Ho LA, Kay SA (2005). FKF1 F-box protein mediates cyclic degradation of a repressor of CONSTANS in Arabidopsis. Science.

[pcu181-B24] Inoue I, Nishida H, Okumoto Y, Tanisaka T (1998). Identification of an early heading time gene found in the Taiwanese rice cultivar Taichung. Breed. Sci..

[pcu181-B25] Itoh H, Izawa T (2013). The coincidence of critical day length recognition for florigen gene expression and floral transition under long-day conditions in rice. Mol. Plant.

[pcu181-B26] Itoh H, Nonoue Y, Yano M, Izawa T (2010). A pair of floral regulators sets critical day length for Hd3a florigen expression in rice. Nat. Genet..

[pcu181-B27] Jang S, Marchal V, Panigrahi KC, Wenkel S, Soppe W, Deng XW (2008). Arabidopsis COP1 shapes the temporal pattern of CO accumulation conferring a photoperiodic flowering response. EMBO J..

[pcu181-B28] Kardailsky I, Shukla VK, Ahn JH, Dagenais N, Christensen SK, Nguyen JT (1999). Activation tagging of the floral inducer FT. Science.

[pcu181-B29] Kim J, Kim Y, Yeom M, Kim JH, Nam HG (2008). FIONA1 is essential for regulating period length in the Arabidopsis circadian clock. Plant Cell.

[pcu181-B30] Kloosterman B, Abelenda JA, Gomez Mdel M, Oortwijn M, de Boer JM, Kowitwanich K (2013). Naturally occurring allele diversity allows potato cultivation in northern latitudes. Nature.

[pcu181-B31] Kobayashi Y, Kaya H, Goto K, Iwabuchi M, Araki T (1999). A pair of related genes with antagonistic roles in mediating flowering signals. Science.

[pcu181-B32] Kojima S, Takahashi Y, Kobayashi Y, Monna L, Sasaki T, Araki T (2002). Hd3a, a rice ortholog of the Arabidopsis FT gene, promotes transition to flowering downstream of Hd1 under short-day conditions. Plant Cell Physiol..

[pcu181-B33] Koo BH, Yoo SC, Park JW, Kwon CT, Lee BD, An G (2013). Natural variation in OsPRR37 regulates heading date and contributes to rice cultivation at a wide range of latitudes. Mol. Plant.

[pcu181-B34] Larson G, Piperno DR, Allaby RG, Purugganan MD, Andersson L, Arroyo-Kalin M (2014). Current perspectives and the future of domestication studies. Proc. Natl Acad. Sci. USA.

[pcu181-B35] Liew LC, Hecht V, Laurie RE, Knowles CL, Vander Schoor JK, Macknight RC (2009). DIE NEUTRALIS and LATE BLOOMER 1 contribute to regulation of the pea circadian clock. Plant Cell.

[pcu181-B36] Liew LC, Hecht V, Sussmilch FC, Weller JL (2014). The pea photoperiod response gene STERILE NODES is an ortholog of LUX ARRHYTHMO. Plant Physiol..

[pcu181-B37] Liu LJ, Zhang YC, Li QH, Sang Y, Mao J, Lian HL (2008). COP1-mediated ubiquitination of CONSTANS is implicated in cryptochrome regulation of flowering in Arabidopsis. Plant Cell.

[pcu181-B38] Liu T, Carlsson J, Takeuchi T, Newton L, Farre EM (2013). Direct regulation of abiotic responses by the Arabidopsis circadian clock component PRR7. Plant J..

[pcu181-B39] Matsubara K, Ogiso-Tanaka E, Hori K, Ebana K, Ando T, Yano M (2012). Natural variation in Hd17, a homolog of Arabidopsis ELF3 that is involved in rice photoperiodic flowering. Plant Cell Physiol..

[pcu181-B40] McClung CR (2014). Wheels within wheels: new transcriptional feedback loops in the Arabidopsis circadian clock. F1000Prime Rep..

[pcu181-B41] Mizoguchi T, Wright L, Fujiwara S, Cremer F, Lee K, Onouchi H (2005). Distinct roles of GIGANTEA in promoting flowering and regulating circadian rhythms in Arabidopsis. Plant Cell.

[pcu181-B42] Mizuno N, Nitta M, Sato K, Nasuda S (2012). A wheat homologue of PHYTOCLOCK 1 is a candidate gene conferring the early heading phenotype to einkorn wheat. Genes Genet. Syst..

[pcu181-B43] Murphy RL, Klein RR, Morishige DT, Brady JA, Rooney WL, Miller FR (2011). Coincident light and clock regulation of pseudoresponse regulator protein 37 (PRR37) controls photoperiodic flowering in sorghum. Proc. Natl Acad. Sci. USA.

[pcu181-B44] Murphy RL, Morishige DT, Brady JA, Rooney WL, Yang S, Klein PE (2014). Ghd7 (Ma6) represses sorghum flowering in long days: alleles enhance biomass accumulation and grain production. Plant Genome.

[pcu181-B45] Nakamichi N (2011). Molecular mechanisms underlying the Arabidopsis circadian clock. Plant Cell Physiol..

[pcu181-B46] Nakamichi N, Kiba T, Kamioka M, Suzuki T, Yamashino T, Higashiyama T (2012). Transcriptional repressor PRR5 directly regulates clock-output pathways. Proc. Natl Acad. Sci. USA.

[pcu181-B47] Nakamichi N, Kita M, Niinuma K, Ito S, Yamashino T, Mizoguchi T (2007). Arabidopsis clock-associated pseudo-response regulators PRR9, PRR7 and PRR5 coordinately and positively regulate flowering time through the canonical CONSTANS-dependent photoperiodic pathway. Plant Cell Physiol..

[pcu181-B48] Nelson DC, Lasswell J, Rogg LE, Cohen MA, Bartel B (2000). FKF1, a clock-controlled gene that regulates the transition to flowering in Arabidopsis. Cell.

[pcu181-B49] Ogiso-Tanaka E, Matsubara K, Yamamoto S, Nonoue Y, Wu J, Fujisawa H (2013). Natural variation of the RICE FLOWERING LOCUS T 1 contributes to flowering time divergence in rice. PLoS One.

[pcu181-B50] Onai K, Ishiura M (2005). PHYTOCLOCK 1 encoding a novel GARP protein essential for the Arabidopsis circadian clock. Genes Cells.

[pcu181-B51] Pin PA, Zhang W, Vogt SH, Dally N, Büttner B, Schulze-Buxlih G (2012). The role of a pseudo-response regulator gene in life cycle adaptation and domestication of beet. Curr. Biol..

[pcu181-B52] Putterill J, Robson F, Lee K, Simon R, Coupland G (1995). The CONSTANS gene of Arabidopsis promotes flowering and encodes a protein showing similarities to zinc finger transcription factors. Cell.

[pcu181-B53] Rawat R, Takahashi N, Hsu PY, Jones MA, Schwartz J, Salemi MR (2011). REVEILLE8 and PSEUDO-REPONSE REGULATOR5 form a negative feedback loop within the Arabidopsis circadian clock. PLoS Genet..

[pcu181-B54] Ream TS, Woods DP, Amasino RM (2012). The molecular basis of vernalization in different plant groups. Cold Spring Harb. Symp. Quant. Biol..

[pcu181-B55] Rodriguez-Falcon M, Bou J, Prat S (2006). Seasonal control of tuberization in potato: conserved elements with the flowering response. Annu. Rev. Plant Biol..

[pcu181-B56] Rosas U, Mei Y, Xie Q, Banta JA, Zhou RW, Suerfferheld G (2014). Variation in Arabidopsis flowering time associated with cis-regulatory variation in CONSTANS. Nat. Commun..

[pcu181-B57] Rugnone ML, Faigon Soverna A, Sanchez SE, Schlaen RG, Hernando CE, Seymour DK (2013). LNK genes integrate light and clock signaling networks at the core of the Arabidopsis oscillator. Proc. Natl Acad. Sci. USA.

[pcu181-B58] Saito H, Ogiso-Tanaka E, Okumoto Y, Yoshitake Y, Izumi H, Yokoo T (2012). Ef7 encodes an ELF3-like protein and promotes rice flowering by negatively regulating the floral repressor gene Ghd7 under both short- and long-day conditions. Plant Cell Physiol..

[pcu181-B59] Saito H, Yuan Q, Okumoto Y, Doi K, Yoshimura A, Inoue H (2009). Multiple alleles at Early flowering 1 locus making variation in the basic vegetative growth period in rice (Oryza sativa L.). Theor. Appl. Genet..

[pcu181-B60] Satake A (2010). Diversity of plant life cycles is generated by dynamic epigenetic regulation in response to vernalization. J. Theor. Biol..

[pcu181-B61] Sawa M, Nusinow DA, Kay SA, Imaizumi T (2007). FKF1 and GIGANTEA complex formation is required for day-length measurement in Arabidopsis. Science.

[pcu181-B62] Shindo C, Aranzana MJ, Lister C, Baxter C, Nicholls C, Nordborg M (2005). Role of FRIGIDA and FLOWERING LOCUS C in determining variation in flowering time of Arabidopsis. Plant Physiol..

[pcu181-B63] Shindo C, Lister C, Crevillen P, Nordborg M, Dean C (2006). Variation in the epigenetic silencing of FLC contributes to natural variation in Arabidopsis vernalization response. Genes Dev..

[pcu181-B64] Shindo C, Sasakuma T (2001). Early heading mutants of T. monococcum and As. squarrosa, A- and D-Genome ancestal species of hexaploid wheat. Breed. Sci..

[pcu181-B65] Song J, Irwin J, Dean C (2013a). Remembering the prolonged cold of winter. Curr. Biol..

[pcu181-B66] Song YH, Ito S, Imaizumi T (2013b). Flowering time regulation: photoperiod- and temperature-sensing in leaves. Trends Plant Sci..

[pcu181-B67] Song YH, Smith RW, To BJ, Millar AJ, Imaizumi T (2012). FKF1 conveys timing information for CONSTANS stabilization in photoperiodic flowering. Science.

[pcu181-B68] Sugano S, Andronis C, Ong MS, Green RM, Tobin EM (1999). The protein kinase CK2 is involved in regulation of circadian rhythms in Arabidopsis. Proc. Natl Acad. Sci. USA.

[pcu181-B69] Takahashi Y, Teshima KM, Yokoi S, Innan H, Shimamoto K (2009). Variations in Hd1 proteins, Hd3a promoters, and Ehd1 expression levels contribute to diversity of flowering time in cultivated rice. Proc. Natl Acad. Sci. USA.

[pcu181-B70] Turner A, Beales J, Faure S, Dunford RP, Laurie DA (2005). The pseudo-response regulator Ppd-H1 provides adaptation to photoperiod in barley. Science.

[pcu181-B71] Valverde F, Mouradov A, Soppe W, Ravenscroft D, Samach A, Coupland G (2004). Photoreceptor regulation of CONSTANS protein in photoperiodic flowering. Science.

[pcu181-B72] Weller JL, Liew LC, Hecht VFG, Rajandran V, Laurie RE, Ridge S (2012). A conserved molecular basis for photoperiod adaptation in two temperate legumes. Proc. Natl Acad. Sci. USA.

[pcu181-B73] Weller JL, Reid JB, Taylor SA, Murfet IC (1997). The genetic control of flowering in pea. Trends Plant Sci.

[pcu181-B74] Worland AJ, Borner A, Korzun V, Li WM, Petrovic S, Sayers EJ (1998). The influence of photoperiod genes on the adaptability of European winter wheats (Reprinted from Wheat: Prospects for global improvement, 1998). Euphytica.

[pcu181-B75] Wu JF, Wang Y, Wu SH (2008). Two new clock proteins, LWD1 and LWD2, regulate Arabidopsis photoperiodic flowering. Plant Physiol..

[pcu181-B76] Xue W, Xing Y, Weng X, Zhao Y, Tang W, Wang L (2008). Natural variation in Ghd7 is an important regulator of heading date and yield potential in rice. Nat. Genet..

[pcu181-B77] Yamamoto T, Kuboki Y, Lin SY, Sasaki T, Yano M (1998). Fine mapping of quantitative trait loci Hd-1, Hd-2 and Hd-3, controlling heading date of rice, as single Mendelian factors. Theor. Appl. Genet..

[pcu181-B78] Yan L, Loukoianov A, Blechl A, Tranquilli G, Ramakrishna W, SanMiguel P (2004). The wheat VRN2 gene is a flowering repressor down-regulated by vernalization. Science.

[pcu181-B79] Yan L, Loukoianov A, Tranquilli G, Helguera M, Fahima T, Dubcovsky J (2003). Positional cloning of the wheat vernalization gene VRN1. Proc. Natl Acad. Sci. USA.

[pcu181-B80] Yan W, Liu H, Zhou X, Li Q, Zhang J, Lu L (2013). Natural variation in Ghd7.1 plays an important role in grain yield and adaptation in rice. Cell Res..

[pcu181-B81] Yang Q, Li Z, Li W, Ku L, Wang C, Lu L (2013). CACTA-like transposable element in ZmCCT attenuated photoperiod sensitivity and accelerated the postdomestication spread of maize. Proc. Natl Acad. Sci. USA.

[pcu181-B82] Yang Y, Peng Q, Chen GX, Li XH, Wu CY (2013). OsELF3 is involved in circadian clock regulation for promoting flowering under long-day conditions in rice. Mol. Plant.

[pcu181-B83] Yano M, Katayose Y, Ashikari M, Yamanouchi U, Monna L, Fuse T (2000). Hd1, a major photoperiod sensitivity quantitative trait locus in rice, is closely related to the Arabidopsis flowering time gene CONSTANS. Plant Cell.

[pcu181-B84] Yanovsky MJ, Kay SA (2002). Molecular basis of seasonal time measurement in Arabidopsis. Nature.

[pcu181-B85] Zakhrabekova S, Gough SP, Braumann I, Muller AH, Lundqvist J, Ahmann K (2012). Induced mutations in circadian clock regulator Mat-a facilitated short-season adaptation and range extension in cultivated barley. Proc. Natl Acad. Sci. USA.

[pcu181-B86] Zhao J, Huang X, Ouyang X, Chen W, Du A, Zhu L (2012). OsELF3-1, an ortholog of Arabidopsis early flowering 3, regulates rice circadian rhythm and photoperiodic flowering. PLoS One.

[pcu181-B87] Zohary D, Hopf M, Weiss E (2011). Domestication of Plants in the Old World.

